# Using GPCRs as Molecular Beacons to Target Ovarian Cancer with Nanomedicines

**DOI:** 10.3390/cancers14102362

**Published:** 2022-05-10

**Authors:** Riya Khetan, Cintya Dharmayanti, Todd A. Gillam, Eric Kübler, Manuela Klingler-Hoffmann, Carmela Ricciardelli, Martin K. Oehler, Anton Blencowe, Sanjay Garg, Hugo Albrecht

**Affiliations:** 1 Clinical and Health Sciences, University of South Australia, Adelaide, SA 5001, Australia; khery005@mymail.unisa.edu.au (R.K.); manuela.klingler-hoffmann@unisa.edu.au (M.K.-H.); 2 Applied Chemistry and Translational Biomaterials Group, Clinical and Health Sciences, University of South Australia, Adelaide, SA 5000, Australia; cintya.dharmayanti@unisa.edu.au (C.D.); todd.gillam@unisa.edu.au (T.A.G.); anton.blencowe@unisa.edu.au (A.B.); 3 Institute for Chemistry and Bioanalytics, School of Life Sciences, University of Applied Sciences and Arts Northwestern Switzerland FHNW, 4132 Muttenz, Switzerland; eric.kuebler@fhnw.ch; 4 Discipline of Obstetrics and Gynecology, School of Medicine, Robinson Research Institute, University of Adelaide, Adelaide, SA 5005, Australia; carmela.ricciardelli@adelaide.edu.au (C.R.); martin.oehler@adelaide.edu.au (M.K.O.); 5 Department of Gynecological Oncology, Royal Adelaide Hospital, Adelaide, SA 5000, Australia; 6 Centre for Pharmaceutical Innovation, Clinical and Health Sciences, University of South Australia, Adelaide, SA 5001, Australia

**Keywords:** ovarian cancer, G protein-coupled receptor, GPCR, nanoparticle, active targeting, drug delivery, receptor, internalization, ligand

## Abstract

**Simple Summary:**

The five-year survival rate for ovarian cancer is less than 50%, resulting in a global burden of >140,000 deaths annually. Late detection, cancer heterogeneity, and recurrent disease all contribute to treatment failure. Herein, recent advancements in the targeted delivery of therapeutics to ovarian cancer using nanoparticles are reviewed. In addition, we explore the applicability of targeting highly expressed cell surface receptors in ovarian cancer tissue to direct drug-loaded nanoparticle delivery systems. Targeted nanomedicine strategies have the potential to increase drug accumulation in tumor cells, prevent adverse effects on healthy tissue and lead to improved patient outcomes.

**Abstract:**

The five-year survival rate for women with ovarian cancer is very poor despite radical cytoreductive surgery and chemotherapy. Although most patients initially respond to platinum-based chemotherapy, the majority experience recurrence and ultimately develop chemoresistance, resulting in fatal outcomes. The current administration of cytotoxic compounds is hampered by dose-limiting severe adverse effects. There is an unmet clinical need for targeted drug delivery systems that transport chemotherapeutics selectively to tumor cells while minimizing off-target toxicity. G protein-coupled receptors (GPCRs) are the largest family of membrane receptors, and many are overexpressed in solid tumors, including ovarian cancer. This review summarizes the progress in engineered nanoparticle research for drug delivery for ovarian cancer and discusses the potential use of GPCRs as molecular entry points to deliver anti-cancer compounds into ovarian cancer cells. A newly emerging treatment paradigm could be the personalized design of nanomedicines on a case-by-case basis.

## 1. Introduction

The symptoms of ovarian cancer are non-specific, and due to a lack of effective screening strategies, it is generally not detected at an early stage when the tumor is still confined to the organ of origin. Resultingly, 70% of patients are diagnosed when their ovarian cancer has progressed to advanced stages (III or IV). The epidemiology of ovarian cancer is very complex and is affected by various factors, including the status of inherited and acquired somatic mutations, hormonal effects during menopause, environmental hazards, pelvic inflammatory disease, endometriosis, and polycystic ovarian syndrome [1]. Effective disease management is extremely challenging, with an overall 5-year survival rate of less than 50%, in comparison to 91% for breast cancer. Only 25% of women with advanced stage ovarian cancer will survive for longer than 5 years.

The current frontline treatment for ovarian cancer includes debulking surgery, followed by dual carboplatin plus paclitaxel chemotherapy to kill residual tumor tissue [2]. The drugs are generally administered intravenously, or according to some treatment protocols, intraperitoneally (IP) [3]. In some patients, good results have also been achieved via a neoadjuvant approach, providing chemotherapy prior to surgical intervention. Whilst approximately 80% of patients who receive the first-line standard treatment of carboplatin plus paclitaxel will respond, often with apparent complete remission, the majority of patients relapse, and about 20% of patients do not respond to the treatment at all, meaning they possess an innate form of chemotherapy resistance [4,5]. Moreover, the currently used treatments are associated with severe and dose-limiting adverse effects. 

To achieve treatments with improved therapeutic indices, several specific molecular therapies have been introduced into the clinic. Poly ADP-ribose polymerase (PARP) inhibition is now used as a strategy against tumors with germline or somatic BRCA1/2 and other DNA repair gene mutations [6,7]. Olaparib was the first PARP inhibitor to gain FDA approval and was followed by others, such as Rucaparib and Niraparib [8]. Administration of these inhibitors can cause severe and dose-limiting hematologic adverse effects, such as thrombocytopenia, neutropenia, and anemia [8]. Grade 3/4 toxicities have been reported to force dose interruptions and reductions [9]. Apart from other mild side effects, increased frequencies of myeloblastic syndromes and acute myeloid leukemia have also been observed [10]. Another treatment strategy employs Bevacizumab as antibody-based, anti-angiogenic therapy, as maintenance therapy in the adjuvant setting, or in combination with other chemotherapy drugs such as liposomal doxorubicin or gemcitabine at recurrence [11]. An alternative anti-angiogenic is the kinase inhibitor Cediranib, an inhibitor of VEGFR-1, -2, -3, and c-kit. Additional novel therapies under investigation include vaccines [12], CAR-T immunotherapy, anti CA125 antibody therapy, and viral and small molecule immune checkpoint inhibitors [13,14,15,16,17,18].

In summary, limited progress has been made in providing new first-line ovarian cancer treatments over the last 30 years, and there remains an urgent unmet clinical need to develop better treatment regimens. To achieve these objectives, it is imperative that the tumor architecture, including the tumor microenvironment and tumor-associated genomic/proteomic landscape, is well understood. The latter can shed light on specifically overexpressed cell surface receptors, guiding the rational design of next-generation nanoparticle (NP)-based formulations, exploiting receptor-mediated cellular uptake to achieve tumor-specific drug delivery. In light of this, the potential of GPCRs as targeting receptors for future ovarian cancer nanomedicines is presented herein.

## 2. Tissue Architecture of Ovarian Tumors

Epithelial ovarian cancer (EOC) comprises about 90% of all ovarian tumors and is subdivided into five major types, including high-grade and low-grade serous ovarian carcinoma (HGSOC and LGSOC), endometrioid carcinoma, clear cell carcinoma, and mucinous carcinoma [19]. HGSOC comprises approximately 75% of all EOCs and therefore, our discussion is largely focused on this subtype of ovarian cancer.

### From Primary Tumors to Ascites to Metastases

**Figure 1:**
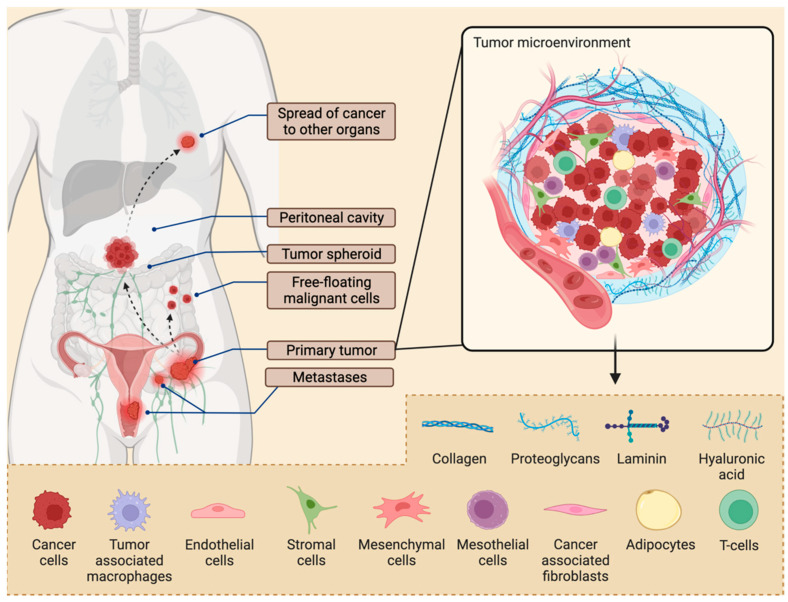
Ovarian cancer metastasis and tumor architecture. Created with BioRender.com.

Malignant cells most frequently develop in the lining of the fallopian tubes, and sometimes on the surface of the ovaries or peritoneum. Ovarian cancers gain immediate access to the abdominal cavity when malignant cells detach from the primary tumor and survive in the peritoneal cavity as tumor spheroids or free-floating malignant cells. This process is accompanied by an epithelial to mesenchymal transition (EMT), driving the shedding of tumor cells into the peritoneal space [20,21], followed by a mesenchymal-to-epithelial transition (MET) allowing attachment to mesothelial cells and subsequently leading to colonization of serous membranes and the formation of secondary lesions [22,23,24,25,26]. Malignant ascites are prevalent in over one-third of patients diagnosed with FIGO stage III and IV disease, and in almost all patients with tumor recurrences [27]. The occurrence of malignant ascites correlates with poor prognosis. The tumor mass and ascites consist of a mixture of cancer, immune and stromal cells, mainly macrophages, T-cells, fibroblasts, adipocytes, endothelial, mesothelial, and mesenchymal cells, as well as extracellular matrix (ECM) components (Figure 1).

Macrophages contribute to a tumor-friendly environment that induces angiogenesis, supports metastatic growth on the omentum, and supports chemoresistance [28,29,30]. In solid tumors, these effects are further enhanced by neighboring adipocytes, which attract cancer cells through IL-8 and further boost proliferation through the secretion of fatty acids [31]. Tumor-associated macrophages (TAMs) consist of the M-1 type with anti-tumor activities, and the M-2 type with pro-tumor activities. The latter is stimulated by IL-4, -10, and -13 secreted from tumor cells [32]. M-2 macrophages are a major component in ovarian cancer known to suppress T-cell proliferation and boost immune suppression via T_reg_ cell activation [33]. These cells express CTLA-4 and PD-1 receptors, therefore inhibiting cytotoxic functions [34]. M2 cells are further involved in tissue repair, ECM remodeling, and angiogenesis. Another immunosuppressive cell population, myeloid-derived suppressor cells (MDSCs), act in synergy with M2 TAMs to block natural killer cells and cytotoxic T-cells, resulting in cancer cell immune escape [30,35,36,37].

Ovarian cancer cells have been described to induce apoptosis in dendritic cells and lymphocytes [38]. Furthermore, within the tumor environment, fibroblasts can be turned into cancer-associated fibroblasts (CAFs), which release inflammatory factors affecting the ECM, subsequently leading to EMT, proliferation, invasion, chemoresistance, and suppression of apoptosis [39,40,41,42]. Mesenchymal stem cells (MSCs) can transform into fibroblasts, osteocytes, or adipocytes within the tumor microenvironment. In addition to this, MSCs release TGF β, boosting the proliferation of cancer stem cells (CSCs), therefore driving chemoresistance. Ovarian CSCs constitute a small sub-population of central importance during primary tumor initiation, formation of metastasis, and driving resistance to chemotherapy [43,44]. Regarding pro-metastatic factors, TAMs, CAFs, and adipocytes boost angiogenesis via the release of VEGF. The effects on endothelial cells are mediated by enhanced survival, proliferation, migration, and vascular permeability [45].

Finally, the tumor microenvironment is complemented by the ECM, which is made up of a mesh of proteins (e.g., collagens, laminin, tenascin, fibronectin), hyaluronan, and various enzymes (e.g., matrix metalloproteinases, lysyl oxidase, etc.) [46]. Medicines need to be delivered across this fibrous environment that surrounds spheroids and secondary peritoneal metastases. On top of this, a mucus barrier, mainly consisting of water and high molecular weight glycoproteins, the mucins, needs to be overcome to achieve effective drug delivery.

Ovarian tumors are spatially confined to the peritoneal cavity by a serosal exchange surface which comprises approximately 1.5 m^2^ [47]. Hence, IP drug delivery is an appealing route toward using engineered NPs without the need for systemic administration. Once metastatic growths break through the serous membrane, cancer cells can spread into various pelvic organs. Endothelial cells become gradually more essential to vascularize the tissue, when tumor metastases grow larger than approximately 2 mm^3^, at a threshold where cells in newly forming tumor cores will reach a state of hypoxia. Solid tumors are generally associated with compromised vascular integrity and impaired lymphatic drainage; culminating in a phenomenon termed the enhanced permeability and retention (EPR) effect [48]. The EPR effect is a passive mechanism and leads to NP accumulation in solid tumor tissue, as further discussed in Section 3.2 of this review. However, at this stage of cancer progression, peritoneally delivered NPs will reach target cells in the ascites through convection, and advanced metastases through the EPR effect. Hence, it can be speculated that the suggested route will be sufficient without the need for systemic IV administration.

## 3. Currently Used Nanomedicines for Cancer Treatment

Most standard first-line cancer drugs affect growing cells without discrimination between cancerous and healthy cells, causing severe side effects including gastrointestinal reactions, fatigue, hair loss, and bone marrow suppression [49]. Further, many of these drugs typically exhibit narrow therapeutic indices [50], limited by increased drug detoxification and multi-drug resistance [51]. NP drug delivery platforms possess advanced properties which drastically increase treatment efficacy [52], and diminish adverse effects [53,54], overcoming many of the limitations of classic chemotherapeutics.

### 3.1. Advantages of Nanoparticle Drug Delivery

Due to their unique chemical structures and physical properties, certain NPs can improve the stability and biocompatibility of encapsulated drugs and can allow controlled drug release inside tumor cells [55]. They have been demonstrated to significantly enhance the apparent aqueous solubility of hydrophobic drugs and facilitate their absorption [56,57]. For instance, micelles, which are typically formed from amphiphilic compounds (such as block copolymers or surfactants), can self-assemble in aqueous environments to generate nanostructures with a hydrophobic ‘core’ and hydrophilic ‘corona’. The core can encapsulate lipophilic drugs, while the hydrophilic corona can interact with the surrounding water, thereby creating a favorable environment for drug solubilization. Liposomal formulations enhance drug solubility using a similar principle, whereby hydrophobic interactions result in the formation of a bilayer membrane decorated with hydrophilic moieties at the exterior and interior surfaces of the NP. Drugs can either be incorporated within the hydrophobic membrane or into the hydrophilic core, depending on their polarity [58]. Other NPs, such as dendrimers, exploit their hyperbranched structure to incorporate molecules both, at the periphery as well as within their dendritic architecture, to improve drug solubility [59,60]. This enhanced drug solubility can result in improved drug absorption and bioavailability in vivo [61].

Another advantage of encapsulating drugs into NP systems is their ability to provide protection from degradation, particularly from chemical or enzymatic processes [62]. This is particularly important for acid-labile drugs, peptides, proteins, and DNA/RNA payloads, which are especially susceptible to degradation by acidic environments and by proteolytic or nucleolytic enzymes. NP drug delivery can also reduce drug-associated toxicities by minimizing drug accumulation in off-target tissues and organs [53,63].

Additionally, recent design efforts have been directed toward the development of NP systems that feature stimuli-responsive drug release; triggered by light, redox reactions, temperature, or pH; further enhancing spatial or temporal drug release [64,65,66,67,68]. pH-responsivity is particularly advantageous in the context of cancer treatment, as the tumor milieu exhibits a characteristically lower pH (6.5–7.2) than physiological pH (7.4) due to lactic acid production [69]. Similarly, redox-sensitive NPs can exploit the increased levels of intracellular glutathione that are associated with malignancy for drug release [66,70].

### 3.2. Passive Targeting of Nanoparticle Drug Delivery Systems

NP drug delivery systems for the treatment of cancer typically range in size from 20 to 200 nm, which is critical to their passive targeting behavior via the EPR effect [71]. Too small NP dimensions lead to rapid renal excretion, whilst larger, or opsonin decorated NPs are rapidly cleared from the blood stream by phagocytosis of macrophages in the reticuloendothelial system (RES) [72,73,74,75,76]. The interaction of NPs with the RES is determined by factors such as shape, composition, surface charge, and size of the material covering the NPs [72]. It is vital for the NPs to remain undetected by the RES while traveling through the body to successfully deliver their payload to tumor cells. NPs decorated with poly (ethylene glycol) (PEG) can exhibit a steric barrier that reduces protein binding, allowing PEGylated NPs to evade recognition and destruction by the RES and prolonging circulation of the drug (i.e., stealth characteristics) [77,78]. Noteworthy, mucus penetrating properties have been shown with NPs using high PEG saturation on the surface, a property of particular interest in the context of IP delivery for ovarian cancer treatment [79].

### 3.3. Approved Formulations

Several attempts have been undertaken to develop nanomedicines to overcome the associated limitations of classical treatments, but few NP formulations have FDA approval (Table 1). These NPs are generally administered intravenously, relying on EPR-driven tumor targeting. Organic NPs, including liposomal and polymeric formulations, are heavily represented among the approved nanomedicines (Table 1). Liposomal drug delivery platforms are the most prevalent carriers of cancer drugs [80]. For example, Doxil (PEGylated) and Myocet were among the first FDA-approved liposomal nanomedicines carrying the drug doxorubicin (DOX), leading to reduced cardiotoxicity and an extended half-life in the systemic circulation [81,82]. Similarly, albumin-bound paclitaxel (Abraxane) was the first polymeric NP using passive targeting to reach the tumor tissue. A variety of drug-delivery platforms based on other NPs are used in approved nanomedicines such as inorganic NPs and micelles (Table 1).

Despite the promising effects of the FDA-approved NPs, there are still ongoing challenges associated with the toxicity and efficacy of these nano formulations. For instance, liposomal DOX has been shown to induce mucositis and palmar plantar erythrodysesthesia as side effects, which were not observed with earlier classic DOX formulations [83]. Furthermore, most approved NP delivery systems do not feature suitable stimuli-responsive characteristics, which leaves them inert to the influence of intracellular stimuli, such as endosomal pH change. Resultantly, following internalization, a significant proportion of the formulation along with the drug cargo is typically degraded within the lysosome [84]. Therefore, future NP generations in clinical or pre-clinical development have been engineered to address this issue.

### 3.4. Nanomedicines in Clinical Development

An overview of nanomedicines at various stages of clinical development is presented in Table 2. Current clinical development also encompasses the advancement of relatively simple NPs into multi-component drug delivery platforms, which mainly includes improvements in polymer and inorganic chemistry. Moreover, synthetic polymers are often used for the synthesis of liposomes and micelles, and in some cases, NPs are simply coated with synthetic polymers to escape protein adsorption [87]. However, there are challenges associated with the clinical translation of nanomedicines. For example, a polymeric formulation of Camptothecin (CRLX101, Table 2) used against ovarian cancer treatment was terminated due to various drawbacks including drug resistance, poor solubility, and stability, as well as unpredictable adverse drug interactions [100]. To improve the therapeutic efficacy of nanomedicines, a more comprehensive understanding of how NP components interact with encapsulated drugs and patient tissues is required. Novel insights may be used to achieve more specific and triggered drug release into cancer tissues, resulting in improved safety and biocompatibility profiles, leading to better clinical trial outcomes [101].

In recent years, NPs have been suggested to enter solid tumors by active processes through epithelial cells, rather than extravasation alone [102]. Elucidating the up-to-date unknown processes by which NPs are actively transported through the epithelial cell layer may expose mechanisms that can be used to make drug targeting more efficient. This can be accomplished through the inclusion of receptor-specific targeting ligands, which facilitate cellular uptake via key internalization pathways that are overactive in malignant states. This confers cell-specific targeting acuity by allowing NPs to interact with receptors that are overexpressed in cancers. Including these advanced attributes in NP drug delivery systems further highlights their utility in the development of enhanced cancer therapeutic platforms.

While all current FDA-approved nanomedicines (Table 1) are based on passive targeting via the EPR effect, the active receptor-mediated targeting approach is gaining traction in the preclinical development arena, where affinity binding of NPs to surface receptors is exploited [87]. SGT-53 (SynerGene Therapeutics), which is in phase one and two trials, is one such example of active targeted delivery for the treatment of glioblastoma, solid tumors, and metastatic pancreatic cancer. The anti-transferrin antibody fragment present in SGT-53 binds to the transferrin glycoprotein receptor on tumor cells [103].

**Table 2:** List of nanomedicines in development.

Carrier System	Nanomedicine (Delivered Drug)	Size (nm)	Targeted Cancer	Status (Recruitment)	Clinical Trial Identifier
Liposomes	ThermoDox (heat-activated) (Doxorubicin)	175	Hepatocellular carcinoma and recurring chest wall breast cancer	Phase III [104] (Completed)	NCT00617981
	Lipoplatin (Cisplatin)	30–80	Pancreatic/head and neck/breast cancer	Phase I [105] (Completed)	NCT00703638
	Lipoxal (Oxaliplatin)	32–56	Advanced cancers	Phase I [106] (Completed)	NCT00355888
	Alocrest (Vinorelbine)	100	Solid tumors	Phase I [107] (Unknown)	NCT00006088
	Lipocurc (Curcumin)	115–120	Advanced cancer	Phase I/II [108] (Unknown)	NCT02138955
	L-Annamycin (Annamycin)	150–188	Acute lymphocytic leukemia	Phase I/II [109] (Unknown)	NCT00271063
	Promitil (Mitomycin-C)	95–100	Advanced solid tumors	Phase I [110] (Completed)	NCT03823989
	Nanobins (Arsenic trioxide)	100	Acute Promyelocytic Leukemia, ovarian and endometrial cancer	Phase II [111] (Recruiting)	NCT03624270 NCT04489706
	LEP-ETU (Paclitaxel)	150	Ovarian/breast/lung cancers	Phase I/II [112] (Completed)	NCT00080418 NCT01190982
	OSI-211 (Lurtotecan)	45–100	Lung cancer/recurrent ovarian	Phase II [113] (Completed)	NCT00046787
	Ceramide nanoliposome (Ceramide)	90	Solid tumor	Phase I [114] (Unknown)	NCT02834611
	Stimuvax (Tecemotide)	150–180	NSCLC, breast, and prostate cancer	Phase III [115] (Terminated)	NCT01423760
	SPI-077 (Cisplatin)	110	Lung, neck, and head cancer	Phase I/II [116] (Completed)	NCT01861496
	Endotag-I (Paclitaxel)	180–200	Breast and pancreatic cancer	Phase II [117] (Completed)	NCT01537536
	MCC-465 (Doxorubicin)	100–145	Stomach cancer	Phase I [118] (Unknown)	-
Albumin	ABI-008 (Docetaxel)	150	Prostate cancer	Phase I/II [119] (Completed)	NCT00477529
	ABI-009 (Rapamycin)	100	Colorectal cancer	Phase I/II [119] (Active, not recruiting)	NCT03439462
Polymeric	CRLX101 (Camptothecin)	20–50	Ovarian cancer	Phase I/II [120] (Terminated)	NCT02389985
	DHAD-PBCA (Mitoxantrone)	49–61	Hepatocellular carcinoma	Phase I [121] (Not recruiting)	NCT04331743
	MTX-HAS (Methotrexate)	123–346	Non-melanoma skin cancer	Phase II/III [122] (Completed)	NCT05315128
	PEG-PCL cyclic ketals (Dexamethasone)	110	Acute lymphoblastic leukemia	Pre-clinical [123] (Recruiting)	NCT03390387
Micelles	Paclical (Paclitaxel)	20–60	Epithelial ovarian cancer	Phase III [124] (Completed)	NCT00989131
Gold nanoshell	Auroshell	150	Aurolace therapy of cancer Head and neck cancer	Phase I [125] (Completed)	NCT00848042

Overall, the IP route should be considered to target cancer cells in the peritoneum, and novel approaches need to also consider targeting stromal and immune cells within the tumor microenvironment (e.g., M2 macrophages, endothelial, etc.). A combined approach will use the EPR effect to passively accumulate NPs close to tumor sites, while simultaneously targeting overexpressed receptors after breaking through biological barriers. Recent cutting-edge molecular biology and proteomics technologies will help to understand the receptor landscape on tumors and tumor-associated cells and will be crucial to design strategies to target tumor cells, including CSCs, to avoid tumor recurrence.

### 3.5. Drug Delivery Challenges Using NPs

Several challenges exist in the delivery of drugs to cancer cells that hinder their progression into the clinic. Firstly, many conventional chemotherapeutic drugs exhibit an array of toxicities resulting from unwanted interactions with off-target receptors. For example, nausea, one of the most commonly reported side effects of chemotherapy, is thought to be associated with off-target action against receptors such as the 5-hydroxytrypamine-3 (5-HT3), neurokinin-1, and cholecystokinin-1 receptors [126]. Additionally, due to their indiscriminate mechanism of action in causing DNA damage, traditional antineoplastic agents also exhibit off-target damage to rapidly dividing healthy cells, leading to the hair loss commonly observed in cancer patients [127]. Off-target effects have been addressed, in part, through the development of next-generation drugs that target key molecular pathways to enhance drug selectivity, as well as the encapsulation of drugs within NP carriers to increase specific cell uptake. However, many important considerations remain, and the latter approach will be the focus of this discussion.

While the incorporation of anti-cancer drugs into NP carriers has paved the way for controlled drug release, these vehicles can still be susceptible to rapid recognition and sequestration by the RES through the process of opsonization [128]. Opsonin proteins attached to the surface of polymeric NPs can be recognized by phagocytic cells, which causes the NPs to be either renally cleared, or become sequestered in the liver and spleen, which can lead to potential toxicities or adverse effects. To hinder opsonization and premature destruction, NPs can be decorated with neutral, hydrophilic, ‘shielding’ polymer brushes such as PEG, which prevents the electrostatic and hydrophobic interactions typically required by opsonin proteins to bind to the NP surface. However, the PEGylation of NPs can present other issues, particularly when used in conjunction with active targeting ligands at the NP surface. Due to the flexible nature of PEG, the chains can act to ‘mask’ or bury the ligands within the PEG brush, preventing proper ligand presentation and hindering receptor-mediated uptake [129].

Degradation of NPs is another key consideration, as the use of certain non-biodegradable polymers has been reported to result in toxicity [130]. Several biodegradable polymers have since been investigated [131], though their potential degradation products and metabolites must still be taken into account to avoid adverse effects.

Another important issue is the formation of a protein adsorption layer (or “protein corona”) at the NP surface. Proteins are ubiquitous and diverse in biological fluids and can adsorb to NPs to endow them with different biological properties compared to that of the original particle. This is of notable importance, as the presence of a protein corona can impact cellular recognition, NP uptake, and toxicity. For instance, cellular uptake was reduced for silica NPs with a protein corona compared to protein-free NPs [132]. However, the formation of protein coronas, how they may differ between individuals, and their biological implications are grand challenges that still require much research to be fully understood [130,133].

Finally, genetic heterogeneity (both intertumoral and intratumoral) poses major challenges in ovarian cancer treatment and is a driving factor for intrinsic sensitivity or resistance to chemotherapy. Different individuals with tumors of the same histology often demonstrate huge genotypic variations, making it difficult to standardize therapy to a specific tumor type [134]. Genetic heterogeneity can also present intratumorally (within a single lesion) giving rise to tumors containing multiple co-existing cell types. As a result of this genetic heterogeneity, there is a much wider variety of cells requiring elimination for curative therapy and there is no single molecular target that can be exploited for drug delivery using NPs [135]. This highlights the need for a personalized approach to cancer treatment, where specifically overexpressed receptors can be targeted using NPs based on an active receptor targeting approach.

## 4. Novel Nanoparticle Strategies for Active Receptor Targeting

**Figure 2:**
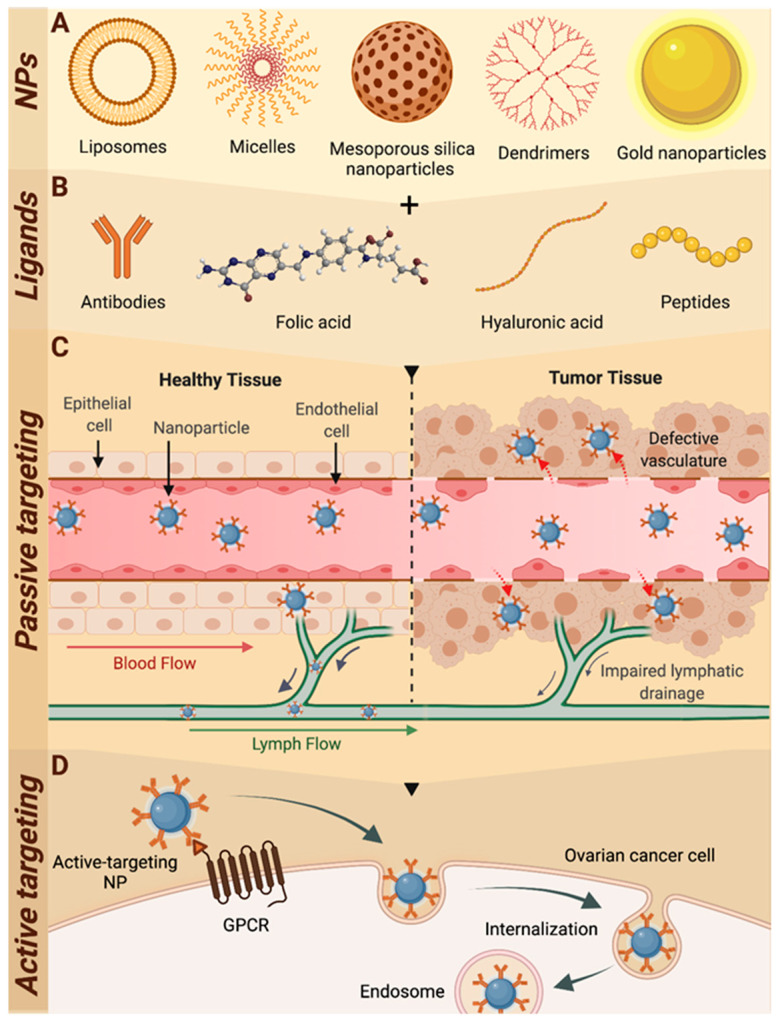
Targeted ovarian cancer NP drug delivery systems: (**A**) diversity of NP drug delivery platform; (**B**) active-targeting ligands of relevance for ovarian cancers (Section 4.1.3); (**C**) passive tumor targeting via the EPR effect; (**D**) internalization of active-targeting NPs driven by receptor activation (e.g., a GPCR or other internalizing receptors). Created with BioRender.com.

A variety of NP drug delivery systems have been developed, each with unique and advantageous structural and physicochemical attributes for the delivery of drugs; including micelles [136,137], dendrimers [138], liposomes [58,139], mesoporous silica [140], and gold NPs [141], among many others. The diversity of NP drug delivery platforms, successful examples of receptor/cell-specific active-targeting strategies relevant to ovarian cancer, and passive tumor targeting behavior are discussed herein and are illustrated in Figure 2.

### 4.1. Active-Targeting Nanoparticles for Ovarian Cancer

A number of receptor-specific ligands have been used to furnish NP drug delivery platforms with active targeting motifs for ovarian cancer. These targeting molecules include bioactive small molecules, hyaluronic acid, peptides/proteins (including antibodies), and steroids. Receptor targeting with these ligand types will be discussed herein.

#### 4.1.1. Bioactive Small Molecules

The coupling of bioactive small molecules that are natural substrates of the target receptor has facilitated the selective uptake of therapeutic-loaded NP assemblies by target cells, by virtue of receptor-mediated internalization pathways.

For example, the overexpression of the folate receptor by ovarian cancer cells provides a viable target for drug delivery to these malignancies. The folate/folic acid system is an established means of developing effective vectors for transit into ovarian cancer cell lines [142,143]. The incorporation of folate for targeted ovarian cancer uptake has been demonstrated in several NP architectures.

Werner et al., utilized this targeting strategy effectively in the development of folate decorated poly (d,l-lactide-*co*-glycolide) (PLGA)-lecithin-PEG core-shell NPs and demonstrated the targeted delivery of chemo- and radio-therapeutics in a murine ovarian intraperitoneal metastasis model [144]. Similarly, the tethering of folate to tri-block copolymer micelles has proven to be an effective platform for siRNA delivery into SKOV3 ovarian cancer cells [145].

Liposomal formulations prepared by Prajapati et al. were furnished with folate, whereby the optimal ligand density was found to be 480 folate moieties per liposome [146]. The folate-labeled liposomes showed more than a 16-fold increase in the area under the curve during in vivo pharmacokinetic studies in rats, as well as a 6.7-fold reduction in tumor volume. Similarly, Wang et al. prepared polymersomes labeled with folate for the delivery of volasertib and PLK1-specific siRNA [147]. The optimal folate density was found to be 20% for SKOV3 cells, which resulted in significantly higher cell uptake as well as higher tumor inhibition in vivo compared to the unlabeled NPs and free drugs.

Dendrimer nanoarchitectures have also been labeled with folate to facilitate active targeting for the folate receptor. For example, Luong et al. developed folate-decorated polyamidoamine (PAMAM) dendrimers for the improved delivery of the anti-cancer flavonoid 3,4-difluorobenzylidene diferuloylmethane into SKOV3 cells [148]. These folate-targeted dendrimers exhibited a significant increase in cellular uptake and cytotoxicity with SKOV3 cells compared to their unlabeled counterparts as a result of receptor-mediated uptake.

A variety of other nanoarchitectures, such as hybrid inorganic glucose/gluconic-acid coated magnetic NPs [149], and gold NPs [150], have also been furnished with folate molecules for improved ovarian cancer targeting.

#### 4.1.2. Hyaluronic Acid

Hyaluronic acid (HA) is a naturally occurring anionic and polymeric glycosaminoglycan, consisting of d-glucuronic acid and *N*-acetyl-d-glucosamine repeat units. It has been readily adopted into nanomedicine as a vehicle for cancer-targeted drug delivery. HA binds to CD44, a ubiquitous cell surface glycoprotein overexpressed in a number of solid tumors, particularly breast, lung, and ovarian cancer [151]. CD44-mediated endocytosis is typically more efficient as the molecular weight of the HAs is increased (e.g., 1000 kDA) [152,153]. As such, HA is often incorporated as a prominent structural component within a nanocarrier, be it as a unimer component in polymeric micelles, as a surface coating of inorganic NPs, or simply in the formation of HA-based nano-gels for drug delivery [152]. HAs are rapidly decomposed by hyaluronidases, providing a mechanism of NP breakdown and drug release which has been a further benefit to HA nanomedicines.

HA has been widely explored as a targeting vector for ovarian cancer drug delivery. For instance, Wang et al. prepared paclitaxel-loaded cationic lipid NPs composed of 1,2-distearoyl phosphatidylethanolamine, which were coated with HA by means of electrostatic adsorption [154]. The anti-tumor efficiency of the HA-coated NPs was evaluated in mice bearing ovarian cancer xenografts and was shown to have a higher tumor inhibition rate in vivo compared to both the uncoated NPs and the free drug. Coating the NPs with HA led to reduced drug accumulation in the heart and kidney, and increased drug concentrations at the tumor site 12–48 h after intravenous injection, highlighting HA as an attractive route towards improved targeting of ovarian cancer tissues [154].

HA-labeled PLGA NPs loaded with paclitaxel and focal adhesion kinase (FAK) siRNA were prepared by Byeon et al. The HA-labelled NPs exhibited higher binding efficiencies for CD44-positive tumor cells, resulting in elevated cytotoxicity and apoptosis in drug-resistant tumor cells, and significantly inhibited tumor growth in patient-derived xenograft models compared to the free drug [155]. Other PLGA-based NPs have also been furnished with HA for CD44 receptor targeting [156].

A variety of additional nanostructures have been coated with HA for CD44 targeting purposes. For instance, Liu et al. prepared coated gold nanorods for targeted photodynamic therapy and chemotherapy [157]. Using layer-by-layer deposition, poly (glutamic acid), DOX, and poly (lysine) were coated onto gold nanorods. HA was applied as a final coating, exploiting the electrostatic interactions between the cationic poly (lysine) and anionic HA, to enable targeting toward CD44 receptors [157]. Shahin et al. prepared HA-conjugated mesoporous silica NPs (MSNs) carrying siRNA against the TWIST protein (siTWIST), which is found to be upregulated in ovarian cancer and plays a key role in cancer metastasis [158]. In the absence of a carrier, the siTWIST was unable to enter the target cells, however, incorporation of the siTWIST into HA-bound MSNs resulted in successful delivery into the target cells within 1 h. The HA-MSNs were found to show significantly greater localization in tumor sites over other tissues and organs compared to untargeted MSNs, emphasizing the tumor-targeting capabilities of HA [158]. Stearic acid (SA)-modified polyethyleneimine (PEI) NPs were covalently conjugated to HA and used as a carrier for ovarian cancer drug delivery [159]. The in vivo distribution of the HA-labelled SA-PEI NPs was investigated in tumor-bearing mice using the near-infrared dye indocyanine green (ICG). It was observed that there was a significant accumulation of the NPs in the tumor compared to other organs such as the heart, kidneys, and spleen, indicating that the HA was effective in targeting the tumor tissue in vivo. However, similarly, high fluorescence intensity was also observed in the liver, which was attributed to a larger liver volume [159]. Other nanostructures, such as iron oxide NPs, have also used HA to target CD44 receptors for ovarian cancer [160,161].

#### 4.1.3. Steroids

Few steroid compounds have been investigated for use as targeting vectors for ovarian cancer NPs. The most prominent example is progesterone, which was used as an active-targeting ligand for casein-calcium ferrite nanohybrid drug carriers, as progesterone receptors are thought to be a marker for ovarian malignancies [162]. Attachment of progesterone to the nanohybrids resulted in selective binding to membrane progesterone receptors, resulting in a >30-fold increase in the anti-cancer potential of hesperidin [162].

#### 4.1.4. Antibodies and Peptides

Targeting specific receptors has often been accomplished with specific antibodies, or through the identification of appropriate peptide and protein ligands. In some cases, peptides have been modified (e.g., the use of cyclic peptides, peptide fragments, and unnatural amino acids) to optimize binding potency, size and biostability. Overall, furnishing NPs with these ligands has proven to be an effective means of attaining highly specific targeting capabilities.

##### Antibodies

Antibodies are another promising avenue by which active targeting has been imparted into NPs for ovarian cancer drug delivery. For instance, Cetuximab, a chimeric monoclonal antibody that binds to the epidermal growth factor receptor (EGFR), was conjugated to cationic gold NPs to target ovarian cancer cells for the purpose of gene delivery [163]. Specific uptake of Cetuximab-labelled NPs was observed in SKOV3 cells, but not Chinese Hamster Ovary (CHO) cells, which was attributed to the lower expression of EGFR in the latter cell line. Significant suppression of tumor growth was observed in vivo for the Cetuximab-labelled NPs compared to the unlabeled control [163].

Another antibody, Trastuzumab, has been used to facilitate active targeting toward the HER2 receptor, which is commonly overexpressed in breast and ovarian cancers. Trastuzumab was conjugated to polymeric micelles for siRNA delivery and demonstrated successful uptake into HER2-overexpressing SKOV3 cells and enhanced tumor localization in vivo [164]. The same antibody was used by Dai et al. as a ligand on the surface of gold NPs to target ErbB2 (also known as HER2) receptors [165]. Interestingly, less than 14 of the 1,000,000 administered NPs were found to interact with the cancer cells. In fact, the majority (~90%) of cell-bound NPs showed uptake into tumor-associated macrophages rather than cancer cells, suggesting that there are complex barriers that exist to achieve active targeting in vivo [165].

##### Peptides

Cyclic RGD containing peptide motifs (cRGD; Arg-Gly-Asp-D-Phe-Cys) have been established to target overexpressed α_v_β_3_ integrin receptors on ovarian cancer cells [166]. cRGD and fluorescent cyanine5 dye were conjugated onto the periphery of multi-arm star block copolymers composed of PAMAM-*block*-poly (aspartic acid)-*block*-PEG, which were loaded with carboplatin [166]. It was found that the cRGD-labeled NPs demonstrated a 3.4-fold increase in cellular uptake in OVCAR3 cells compared to the unlabeled NPs, which was thought to be the result of receptor-mediated endocytosis. Similarly, cRGD was used to impart active-targeting properties to gemcitabine-loaded PLGA NPs [167]. The cRGD-labelled PLGA NPs demonstrated higher toxicity and cellular uptake into SKOV3 cells compared to the unlabeled NPs and the free drug, which was attributed to the binding of the peptide to α_v_β_3_ integrin receptors [167]. Xu et al., modified MSNs with both HA and the RGD peptide for dual receptor targeting. Interestingly, the MSNs furnished with both the HA and RGD peptide ligands exhibited higher cell uptake and anti-cancer effects compared to single-labeled or unlabeled MSNs, suggesting that dual targeting may have a synergistic effect [168].

The RIPL peptide (IPLVVPLRRRRRRRRC) was used to impart active-targeting capabilities to docetaxel-loaded nanolipids to achieve selectivity for hepsin-overexpressing ovarian cancer cell lines, such as SKOV3 [169]. This surface functionalization led to increased cancer cell apoptosis for the RIPL-labelled NPs compared to those that were unlabeled [169].

Zhang et al. prepared PEI-PEG-based NPs targeted toward the follicle-stimulating hormone receptor (FSHR) in ovarian cancer [170]. The targeting ligand used was FP21, a 21-amino acid peptide with the sequence YTRDLVYGDPARPGIQGTGTF, which was designed to interact with specific binding domains of the FSHR. The peptide was constructed using D-amino acids in place of L-amino acids to reduce enzymatic degradation whilst preserving bioactivity. It was shown that the D-FP21-labelled nanocarriers exhibited higher uptake in ovarian cancer cells and enhanced anti-tumor activity in vivo compared to the L-FP21 complexes and unlabeled NPs [170]. Fan et al., conducted similar experiments using QCHCGKCDSDSTDCT as the FSH-derived targeting peptide, conjugated to Maleimide-PEG-PLA and methoxy-PLA NPs [171]. Paclitaxel was used as a cytotoxic agent, where stronger anti-tumor effects and anti-cell proliferation were displayed in vitro when compared to the free drug in a dose- and time-dependent manner. Moreover, an in vivo study demonstrated reduced weight and size of lymph node metastases in a syngeneic ovarian cancer model using NuTu-19 cells in Fischer 344 rats [171].

Luteinizing hormone-releasing hormone (LHRH; pyroGlu-His-Trp-Ser-Tyr-Gly-Leu-Arg-Pro-Gly-NH_2_) is another peptide that has been used to enhance the specific cell uptake of NPs by binding to the LHRH receptor. For example, Lin et al. conjugated LHRH to HA-cystamine-DOX-based NPs, which accumulated at the tumor site more rapidly in vivo compared to LHRH-free NPs, with little to no accumulation in non-target organs [172]. LHRHR-specific peptides have also been grafted to poly(caprolactone)-based NPs for active-targeting purposes [173].

Put together, these examples highlight the breadth and versatility of peptide targeting ligands in achieving selective cell uptake in ovarian cancer. Noteworthy, the last two examples represent the GPCR family, and keeping in mind that there are >500 of these receptors encoded in the human genome, the enormous potential to exploit GPCR-mediated targeting of ovarian cancer is assessed in the next section.

## 5. Harnessing GPCRs to Target Ovarian Cancer Cells with Nanomedicines

Many tumors have been shown to aberrantly express GPCRs and the overwhelming complexity of an underlying signaling network has been discovered [174,175,176,177,178]. The clinical relevance of GPCR expression in the case of ovarian cancer is highlighted by its effects on cell growth, migration, metastasis, invasion, survival, metabolism, and secretion [179,180,181]. Many GPCRs are expressed in stromal, immune, and endothelial cells of ovarian cancer tissue where they play important roles in tumor growth via stimulation of angiogenesis and other mechanisms. In addition to this, β arrestin 2 expression has been associated with impaired prognosis, hence further boosting the role of GPCRs [182]. On one hand, small molecule-mediated modulation of GPCRs presents a potentially rewarding avenue towards novel anti-cancer solutions; whilst on the other hand, and in context with the presented review, the functional overexpression of these receptors lends itself to the targeting of cancer tissues with ligand decorated nanomedicines.

**Figure 3:**
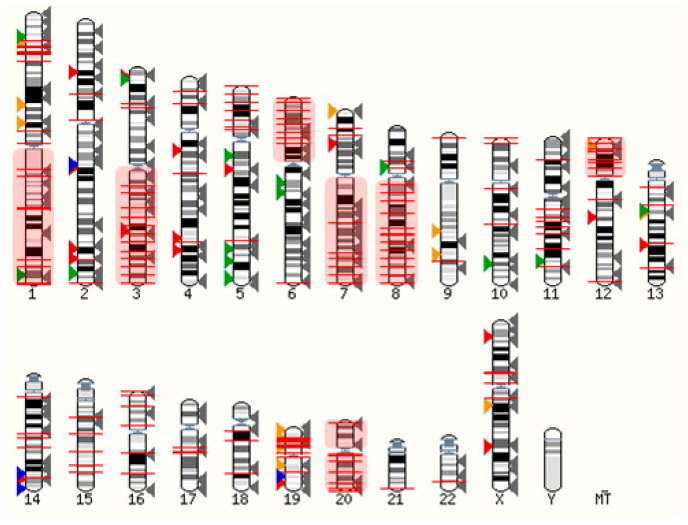
Distribution of GPCRs and copy number alterations (CNA on genome). All GPCRs (except olfactory receptors) are indicated with arrowheads. Overexpressed receptors are shown in color, according to Table 3. Red: Peptide and protein activated receptors; Orange: Lipid receptors; Green: Adrenergic receptors; Blue: Ionic receptors; Grey: Not frequently overexpressed in ovarian cancer (310 receptors). The red lines indicate 2902 CNAs with frequencies ranging from 5%–34% (from TCGA, Pan Cancer Atlas, 572 ovarian serous cystadenocarcinoma samples; accessed via cBioportal [199,200] on 3 March 2022). Frequently amplified chromosome arms 1q, 3q, 6p, 7q, 8q, 12p, 20p, 20q are shown in red shaded boxes. The human karyotype figure was generated using Ensembl 2021 [201].

Ovarian cancers are genetically unstable, most often due to mutations in DNA repair genes (e.g., BRCA1/2) and in the tumor protein P53 gene [183]. The latter is prevalent in ~96% of serous ovarian cancer cells, driving chromosomal instability and leading to aberrant gene expression [184]. Considering that GPCRs are distributed throughout the genome, it is expected that some become frequently overexpressed when healthy cells turn into neoplastic cells. As shown in Figure 3, many GPCR loci are close to copy number alterations (CNAs) or on frequently amplified chromosome arms 1q, 3q, 6p, 7q, 8q, 12p, 20p, and 20q [185]. Although the detailed mechanisms are poorly understood, many GPCRs are very likely to be upregulated within amplicons or as a consequence of chromosomal translocations. Alternatively, the gene expression can be triggered indirectly through upregulated signaling pathways. Whilst some genetic loci may be hotspots for gene amplification and chromosomal translocations, others may be infrequent or at random. Therefore, the basic concept to harness overexpressed GPCRs as molecular entry sites requires a personalized approach, as further discussed in Section 6.

The most studied GPCRs in the context of ovarian cancer are the somatostatin (SSTR1–5) [186,187,188,189], cholecystokinin (CCKAR/CCKBR) [190,191], gastrin-releasing peptide (GRPR) [192,193,194], luteinizing hormone-releasing hormone (LHRHR) [195,196] and neurotensin receptors (NTSR1/2) [197]. Surprisingly, GPCRs have remained largely untapped for targeted drug delivery in cancer tissues. A large body of research reveals many more receptors with the potential to be established as novel biomarkers or docking sites for ligand decorated and drug-loaded NPs (summarized in Table 3). Receptor-mediated internalization will bring NPs directly into endosomal compartments, whereby a pH-responsive drug release trigger can be used to drive NP disassembly, followed by endosomal membrane rupture and drug escape into the cytoplasm, ultimately inducing apoptotic cell death [84,198].

### 5.1. Ionic GPCRs

G-protein coupled receptor 4 (GPR4) is a type of GPCR that is activated by protons and is involved in cancer-related angiogenesis. GPR4 is found to be detected in a higher amount in the endothelium of vessels of EOCs compared to benign ovarian tumors [202]. Bai et al. reported that significant inhibition of invasion and cell growth can be induced in A2780 ovarian cancer cells with the knockdown of GPR4 and transcription factor 7 (TCF7) while promoting apoptosis [203]. Similarly, GPR68 also known as ovarian cancer G protein-coupled receptor 1 (OGR1) has also been identified as a proton sensing receptor [204,205]. The expression of OGR1 in human ovarian tumor HEY cells resulted in the inhibition of cell migration and proliferation [205]. Furthermore, G2A (or GPR132) is also known as a proton sensing receptor that regulates proliferation, immunity, and oncogenesis and exerts antitumorigenic properties via cell cycle arrest at the G2/M stage [206,207,208]. GPR4, GPR68, and GPR132 are recognized to be activated by lysophosphatidylcholine (LPC) and sphingosylphosphorylcholine (SPC), which induces growth inhibition [202,206,209]. Lastly, GPR39 is frequently overexpressed in ovarian cancer tissue and mediates Zn^2+^ induced signaling [210]. GPR39 was found to be an inhibitor of cell death, hence representing a potential therapeutic target for the treatment of ovarian cancer [211].

Most of these receptors do not have suitable ligands to functionalize NPs. However, specific antibodies could be applied to decorate NPs to induce specific binding to the receptor. In the case of proton sensing receptors, endosomal uptake will be triggered in the acidic tumor microenvironment.

### 5.2. Aminergic GPCRs

Aminergic GPCRs, a subset of class A rhodopsin-like GPCRs, are the targets for approximately 25% of the current clinically used drugs [212]. Ovarian cancer is known to be affected by receptor ligands produced by the immune and nervous systems. Receptors from the aminergic GPCR family are excellent drug targets as they are associated with memory, neurotransmission, mood and circadian cycle regulation, cognition, and vasoconstriction [213]. Ovarian cancer is known to be affected by receptor ligands produced by the immune and nervous systems. In line with this, histamine, acetylcholine, serotonin, dopamine, and adrenaline receptors are frequently expressed in ovarian cancer cells and have been linked to their functions including proliferation, survival, and migration [214,215].

Oppitz et al. reported that 23 out of 39 ovarian tumors tested, expressed adrenaline receptors, which was associated with reduced patient survival [216]. Dopamine receptor 2 (DRD2) is known to be overexpressed in ovarian cancer cells. Yong et al. studied the effect of a DRD2 antagonist, thioridazine and it was observed that it exhibited an anticancer effect in A2780 and SKOV3 cell lines as well as SKOV3 xenografts in nude mice by inducing apoptosis and oxidative stress [217]. Moreover, thioridazine interacted with extracellular-signal-regulated kinase (ERK) and AKT signaling pathways and inhibited tumor angiogenesis. Histamine activates the histamine receptor H1 (HRH1), which stimulates the growth of ovarian tumor cells in vitro and promotes the release of extracellular vesicles (EVs) that modulate different steps of the metastatic process. Pyrilamine, a selective HRH1 antagonist can block the cell proliferating effect of histamine on OVCAR3 cells, hence acting as a therapeutic drug target for the death of tumor cells [218].

### 5.3. Lipid GPCRs

Lipids can act as a signaling molecules, store energy, are involved in post-translational modifications, and, lastly, are a major constituent of cellular membranes [219,220,221]. These membrane lipids also play an important role in various tumorigenesis processes such as migration, proliferation, and inflammation [222,223,224]. They are known to directly interact with their targets or bind to extracellular or intracellular receptors. A significant number of lipid-activated GPCRs are known to be expressed in ovarian cancer tissue and their cognate ligands, such as lysophosphatidic acid (LPA), sphingosine-1-phosphate, platelet-activating factor (PAF; 1–0-alkyl-2-acetyl-sn-glycero-3-phosphocholine) and various free fatty acids achieve high local concentrations. Here, we discuss four classes of lipid GPCRs: fatty acid, lysophospholipid, phospholipid, and steroid GPCRs.

#### 5.3.1. Fatty Acid GPCRs

Fatty acids play a vital role in metabolic disorders and inflammation, hence contributing to tumorigenesis [225]. FFAR1 is a free fatty acid (FFA) receptor that has been found to be overexpressed in HGSOCs. FFA-mediated cancer cell growth has been demonstrated, and targeting this receptor is a potential future strategy [226]. Munkarah et al. demonstrated that the high concentration of GW1100, which is an FFAR1 antagonist was able to partially inhibit the proliferation and viability of cancer cell lines in the presence of serum [226]. Moreover, Hopkins et al. studied the function of FFARs in OVCAR3 and SKOV3 cell lines and examined if FFAR agonists affect their proliferation [227]. mRNA expression studies revealed that both the OVCAR3 and SKOV3 cell lines expressed FFAR1, and SKOV3 also expressed FFAR4 in small amounts. Furthermore, the FFAR1 agonist (GW9508) was able to inhibit the proliferation of both cell lines.

#### 5.3.2. Lysophospholipid GPCRs

Lysophospholipids are known to play an essential role in cellular processes, including migration, proliferation, and immune responses [228,229,230]. The most studied lysophospholipids in cancer biology are LPA and sphingosine-1-phosphate (S1P) [231].

The ascitic fluid contains elevated LPA concentrations and ovarian cancer cells are known to produce high levels of LPA [232,233,234]. LPA has been shown to drive ovarian cancer cell migration and invasion [235], activate NF-kB [236] and AP-1 transcription factors [237], increase cyclooxygenase 2 production, and induce metabolic reprogramming of ovarian cancer cells inducing a glycolytic shift via hypoxia-inducible factor 1 activation [233]. Additional pathways to mediate or synergistically act in concert with LPA stimulation are EGFR and other RTKs, and the Hippo/YAP pathway [235,238]. LPA Receptors (LPARs) are widely expressed in normal ovaries but frequently overexpressed in benign tumors and ovarian cancer tissue [232,233]. Particularly, LPAR2 and LPAR3 have been shown to be frequently overexpressed in ovarian cancer cells and tissues [210,234]. Lysophosphatidylethanolamine has been described as an alternative ligand on some LPA receptors inducing a Ca^2+^ signal and boosting cell migration [239,240]. Overall, these accumulated findings make a very strong case for LPA blockage as a potential anti-cancer strategy.

Similarly, S1P plays a vital role in the regulation of angiogenesis, apoptosis, cell growth, and inflammation. S1P controls the invasiveness of epithelial ovarian cancer cells through a complex mechanism involving multiple GPCR pathways, which regulate ECM-proteolysis and attachment of cells [241]. All five of the known S1P receptors might be involved in this complex interplay, and targeting these receptors could be relevant for some anti-cancer strategies. Visentin et al. demonstrated that the S1P-specific monoclonal antibody LT1002 can neutralize S1P by decreasing the systemic level of IL-8, hence, reducing cell survival and proliferation of tumors in a mouse model [242]. Moreover, S1PR antagonists such as VPC44116, VPC23019, and VPC25239 are known to inhibit the invasion and migration of OVCAR3 cells [243]. Hence, the role of S1P in ovarian cancer needs to be further understood to be able to discover new therapeutic strategies for the management of the disease.

#### 5.3.3. Phospholipid GPCRs

PAF binds to the PAF receptor (PTAFR) and is involved in inflammation and platelet aggregation [244]. PTAFR has been shown to activate the EGFR and ERK signaling pathways in ovarian cancer cells, therefore potentially contributing to cancer progression [147]. Yu et al. investigated the effect of WEB2086 (a PTAFR antagonist) in combination with AG1478 (an EGFR inhibitor) on CAOV3 and SKOV3 cell lines and it was observed that the combination significantly inhibited the invasion and proliferation by inducing apoptosis and arresting the cells at the G0/G1 phase [245]. Moreover, Gao et al. studied that PAF increases the stemness of SKOV3 and A2780 cell lines, and the application of Ginkgolide B, which is a PTAFR inhibitor successfully reduced tumor growth [246]. Therefore, targeting PTAFR could be a potential approach for the treatment of ovarian cancer.

#### 5.3.4. Steroid GPCRs

Steroids are typically hydrophobic polycyclic signaling molecules that elicit cellular actions by binding to intracellular nuclear receptors [247]. Androgens, estrogens, mineralocorticoids, progestogens, and glucocorticoids are a few examples of steroid hormones that regulate cellular interactions with nuclear receptors. Estrogen, for instance, transmits signals via G protein-coupled estrogen receptor 1 (GPER1) to activate the EGFR and promote proliferation [248]. Increased membrane estrogen receptor expression has been observed in high-grade serous samples and correlated with impaired prognosis [182]. However, there are controversies in the study of GPER1 and its effect on ovarian cancer. Ignatov et al. reported that the expression of GPER1 was lower in ovarian cancer tissues when compared to benign ovarian tumors [249]. Moreover, the selective GPER1 agonist, G1, was able to suppress the proliferation of SKOV3 and OVCAR3 cell lines. Conversely, Liu et al. demonstrated that 17β-estradiol and G1 induced proliferation of OVCAR5 cell lines [250]. Limited information is available on the impact and role of GPER1 on ovarian cancer, and therefore further studies are required to confirm the tumor-suppressing or proliferating effect of GPER1 before using it as a drug target.

**Table 3:** GPCRs expressed in ovarian cancer.

	Receptor Protein Symbol ^1^	Endogenous Agonists (Signaling ^2^)	Antagonists	References
Ionic	GPR4	Protons (G_s_, G_i/o_, G_q/11_, G_12/13_)	GPR4 antagonist 3b, NE 52-QQ57	[202]
	GPR39	Zn^2+^ (G_q/11_)	-	[210]
	GPR68	Protons (G_i/o_, G_q/11_)	Psychosine	[204,205,251]
	GPR132	Protons (NA ^3^)	Lysophosphatidylcholine	[206,207]
Aminergic	ADRA1B	Adrenaline, Noradrenaline (G_q/11_)	AH 11110, L-765314, Rec 15/2615	[214]
	ADRB1	Adrenaline, Noradrenaline (G_s_)	Acebutolol, Atenolol, Betaxolol	
	ADRB2	Adrenaline, Noradrenaline (G_s_)	Sotalol, Propafenone, Nadolol	
	ADRB3	Adrenaline, Noradrenaline (G_s_)	L-748337, L-748328	
	CHRM3	Acetylcholine (G_q/11_)	Tropicamide, Tolterodine, Oxybutynin	[214]
	DRD1	Dopamine, 5-Hydroxytryptamine, Noradrenaline (G_s_)	Ecopipam, SCH-23390, SKF-83566	[214]
	DRD2	Dopamine (G_i,_ G_i/o_)	ML321, Raclopride, Domperidone	
	HRH1	Histamine (G_q/11_)	Astemizole, Triprolidine, Azelastine	[214,215]
	HTR1A	5-Hydroxytryptamine (G_i/o_)	Robalzotan, WAY-100635	[214]
	HTR1B	5-Hydroxytryptamine (G_i/o_)	GR-55562	
	HTR1D	5-Hydroxytryptamine (G_i/o_)	SB 714786	
	HTR1E	5-Hydroxytryptamine (G_i/o_)	Rauwolscine, Fluspirilene, Metergoline	
	HTR2A	5-Hydroxytryptamine (G_q/11_)	Compund 3b, Ketanserin	
	HTR2B	5-Hydroxytryptamine (G_q/11_)	EGIS-7625, RS-127445, BF-1	
	HTR4	5-Hydroxytryptamine (G_s_)	RS 100235, GR 113808, SB 204070	
Lipid	FFAR1 (GPR40)	docosahexaenoic acid, α-linolenic acid, myristic acid, oleic acid, long chain carboxylic acids (G_q/11_)	GW1100	[226]
	GPER1	17β-estradiol (G_i/o_)	G15, G36	[182]
	LPAR1	LPA (G_i/o_, G_q/11_, G_12/13_)	AM095, ONO-7300243, AM966	[210,232,233,234,235,236,237,238,239,240,252,253]
	LPAR2	LPA, Farnesyl diphosphate, Farnesyl monophosphate (G_i/o_, G_q/11_, G_12/13_)	H2L5186303	
	LPAR3	LPA, Farnesyl diphosphate, Farnesyl monophosphate (Gi_/o_, G_q/11_)	Dioctanoylglycerol pyrophosphate	
	LPAR4	LPA, Farnesyl diphosphate (G_s_, G_i/o_, G_q/11_, G_12/13_)	AM966, Farnesyl diphosphate, Farnesyl monophosphate	
	LPAR5	LPA, Farnesyl diphosphate, Farnesyl monophosphate, *n*-arachidonoylglycine (G_q/11_, G_12/13_)	TCLPA5, AS2717638	
	LPAR6	LPA (G_s_, G_i/o_, G_12/13_)	-	
	PTAFR	PAF, Methylcarbamyl PAF (G_i/o_, G_q/11_)	Rupatadine, Apafant, BN 50739	[254]
	S1PR1	S1P, Dihydrosphingosine 1-phosphate, Sphingosylphosphorylcholine (G_i/o_)	NIBR-0213, W146	[241]
	S1PR2	S1P, Dihydrosphingosine 1-phosphate, Sphingosylphosphorylcholine (G_S_, G_q/11_, G_12/13_)	JTE-013	
	S1PR3	S1P, Dihydrosphingosine 1-phosphate, Sphingosylphosphorylcholine (G_i/o_, G_q/11_, G_12/13_)	TY-52156	
	S1PR4	S1P, Dihydrosphingosine 1-phosphate, Sphingosylphosphorylcholine (G_i/o_, G_12/13_)	CYM-50358	
	S1PR5	S1P, Dihydrosphingosine 1-phosphate, Sphingosylphosphorylcholine (G_i/o_, G_12/13_)	-	
Peptide- and protein-activated receptors	AGTR1	Angiotensin II (G_q/11_, G_i/o_)	Iosartan, Olmesartan, Telmisartan	[236,255]
	AGTR2	Angiotensin II (G_i/o_)	Olodanrigan, PD123319	
	BDKRB2	Bradykinin (G_s_, G_i/o_, G_q/11_)	Anatibant, Icatibant, FR173657	[214]
	CCKAR	CCK-8, -33, -39, -58 (G_q/11_)	Dexloxiglumide, JNJ-17156516, Devazepide	[190,191]
	CCKBR	CCK-4, -8, -33, gastrin-17 (G_q/11_)	Lorglumide, GW-5823, tetronothiodin	
	CXCR1	Interleukin 8 (G_i/o_)	Navarixin, AZD5069	[214]
	CXCR2	Interleukin 8 (G_i/o_)	SX-517, Elubirixin, SB 225002	[256]
	CXCR4	CXCL12 (G_i/o_)	Mavorixafor, T134, Plerixafor	[257]
	EDNRA	Endothelin-1, -2 (G_q/11_)	Macitentan, Ambrisentan, BQ123	[258,259,260,261]
	EDNRB	Endothelin-1, -2, -3 (G_s_, G_i/o_, G_q/11_)	K-8794, IRL 2500, BQ788	
	F2R (PAR1)	Protease activated/Thrombin (G_q/11_)	RWJ-56110, SCH-79797, Vorapaxar	[262]
	F2RL1 (PAR2)	Protease activated/Serine proteases (G_q/11_)	GB88, I-191, AZ8838	[263]
	FPR2	*n*-formyl-methionyl peptides (FMLP) (G_i/o_)	WRWWWW, t-BOC-FLFLF	[264]
	FSHR	Follicle-stimulating Hormone (G_s_)	FSH deglycosylated α/β	[182,265,266]
	GHRHR	Growth Hormone-releasing Hormone (G_s_)	-	[267]
	GNRHR	Type 1 gonadotropin-releasing Hormone (G_q/11_)	Abarelix, Degarelix, Elagolix	[268]
	GRPR	GRP-(14–27), GRP-(18–27), Neuromedin B and C, (G_q/11_)	Bantag-1, PD 168368, AM-37	[192,193,194]
	LGR5 (GPR49)	R-spondin-1, -2, -3, -4 (Wnt)	-	[269,270]
	LHCGR (LHRHR)	Luteinizing hormone, Chorionic gonadotropin (G_s_)	Deglycosylated chorionic gonadotropin	[182,195,196]
	NTSR1	Neurotensin, Large neuromedin *n* (G_q/11_)	Meclinertant, SR142948A	[197,271]
	NTSR2	Neurotensin (G_q/11_)	-	
	OXTR	Oxytocin, Vasopressin (G_q/11_)	Retosiban, SSR126768A, L-372662	[210]
	PTH2R	Parathyroid Hormone (G_s_)	PTHrP-(7–34), TIP39-(7–39)	[210]
	RXFP1	Relaxin-1, -2, -3 (G_s_, G_i/o_)	B-R13/17K H2 relaxin	[272]
	SSTR1	Cortistatin-14, Somatostatin-14, -28 (G_i/o_)	BIM 23454, SRA880	[186,187,188,189]
	SSTR2	Cortistatin-14, -17, Somatostatin 14, -28 (G_i/o_)	BIM 23454, [D-Tyr^8^]CYN 154806, BIM 23627	
	SSTR3	Somatostatin-28, -14, Cortistatin-17 (G_i/o_)	ACQ090, MK-4256	
	SSTR4	Somatostatin-28, -14, Cortistatin-17 (G_i/o_)	PRL-2915, [L-Tyr^8^]CYN 154806, BIM 23454	
	SSTR5	Somatostatin-14, -28, Cortistatin-14, -17 (G_i/o_)	S5A1, BIM 23056	

^1^ According to the European Bioinformatics Institute (EMBL-EBI), the National Center for Biotechnology Information (NCBI), the Protein Information Resource (PIR) and the Swiss Institute for Bioinformatics (SIB); ^2^ guidetopharmacology.org; ^3^ not applicable, coupling unknown.

### 5.4. Peptide- and Protein-Activated GPCRs

Endogenous protein and peptide ligands are known to activate approximately 118 GPCRs in the human body [273]. Some examples of protein-activated GPCRs and their effects on ovarian carcinoma are discussed below.

Endothelin-1 (ET-1) is secreted by endothelial cells, and it acts as a potent vasoconstrictor through activation of the endothelin receptors A and B (EDNRA/B) on smooth muscle cells. Moreover, endothelin receptors are involved in the regulation of cell survival, mitogenesis, angiogenesis, invasion, and epithelial-to-mesenchymal transition (EMT) in malignancies [274]. These receptors stimulate autocrine growth of ovarian carcinoma in vitro and in vivo in response to ET-1 secretion and drive ovarian tumor progression, metastasis, and drug resistance. Various cancer-promoting pathways have been linked to endothelin receptor activation, including β catenin and EGFR signaling. In line with this, the dual EDNRA/B inhibitor macitentan blocked metastatic progression of ovarian cancer cells [258,259,260,261], and zibotentan, a specific EDNRA inhibitor showed synergistic effects on apoptosis and inhibition of ovarian cancer cell invasion, when used in combination with EGFR inhibitors [275,276].

Various protease-activated receptors (PARs) have been implicated in tumor progression. For example, PAR-2 (F2RL1) is overexpressed in some ovarian cancer tissues mainly inducing cell migration, and PAR-1 (F2R) has been found to be abundant in invasive carcinomas, but not in the healthy ovarian epithelium, driving FAK signaling and promoting cancer malignancy [262,263]. F2R activation leads to the expression and secretion of pro-angiogenic chemokines, such as CCL2 (MCP-1), CXCL1 (GRO-α), and CXCL8 (IL-8).

Some chemokine receptors are known to stimulate angiogenesis using paracrine interaction between chemokine receptors expressed on endothelial cells, and chemokines released from ovarian tumor cells [277]. Noteworthy, CXCL8 is known to trigger the CXCR1 and CXCR2 receptors on endothelial cells, inducing cell migration and proliferation [278]. Specific inhibitors have been shown to block CXCL8 mediated cell migration and to synergistically enhance DOX activity [256]. Another chemokine receptor, CXCR4, is overexpressed in human ovarian cancer and its ligand CXCL12 has been shown to be present in ascitic fluid collected from patients with ovarian carcinoma [279,280,281].

Several inflammation-associated receptors are known to exert relevant effects on ovarian cancer cells: bradykinin and several chemokines can trigger intracellular Ca^2+^ signals in ovarian cancer cells [214], and relaxin production is induced by inflammation, activating prooncogenic pathways via the LGR7 (RXFP1) receptor. Furthermore, relaxin/RXFP1 inhibition reduced ovarian cancer cell viability and reversed cisplatin resistance [272]. The expression of another immune modulatory receptor, the formyl-peptide receptor-2 (FPR2), has been detected in EOC tissues and it plays a role in cell migration and invasion [264]. FPR2 detection is correlated with poor prognosis and could be a valuable prognostic marker.

The multifunctional scaffold protein β-arrestin 2 regulates the signal transduction and internalization of activated GPCRs including the luteinizing hormone/choriogonadotropin receptor (LHCGR) and FSHR [282]. High expression levels of β-arrestin 2 were associated with FSHR and LHCGR expression and correlated with impaired prognosis [182]. FSHR elevated gene expression has been frequently detected in patient-derived tumor samples, and it has been suggested as a potential cancer biomarker [265,266].

Several receptors have been found to be upregulated in ovarian cancer tissues, including growth hormone releasing hormone receptor (GHRHR) [283], GRPR [284], leucine-rich repeat-containing G-protein coupled receptor 5 (LGR5) [269,270], oxytocin receptor (OXTR) and parathyroid hormone 2 receptor (PTH2R) [210]. The angiotensin receptors (AGTR1/2) mediated NF-kB transcription factor activation in ovarian cancer cells, indicating functional expression [236].

In summary, it appears that the peptide and protein-activated GPCRs harbor a massive potential for the specific targeting of nanomedicines against ovarian carcinoma.

## 6. Discussion and Conclusions

Independent of the tumor subtype and histologic origin, very similar treatments are routinely applied to treat ovarian cancer. Innovative and disruptive strategies are needed to eradicate ovarian cancer by considering anatomical, microenvironmental, and genetic properties.

In the absence of metastasis beyond the peritoneal space, targeting ascites as front-line therapy or as maintenance therapy is a very promising option for a large majority of patients. IP delivery of NP drug carriers will provide high drug doses directly to ovarian cancer tissue while reducing non-specific uptake into other organs such as the kidney and the liver. The peritoneal-plasma barrier reduces the rate of drug clearance when compared to plasma [285]. This pharmacokinetic advantage will allow increased time for NPs to deliver drugs to cancer cells.

Active targeting of drug-loaded, ligand-functionalized NPs has emerged as a promising approach to accelerate surface binding kinetics and cellular uptake, therefore further reducing effects due to non-specific clearance from the IP compartment. Fluid convection in the IP space will drive NP transport to free-floating malignant cells and spheroids and poorly vascularized peritoneal metastasis. However, access to tumor cells is compromised by the complexity of the tumor microenvironment and by the different mucosa-covered tissues in the peritoneal cavity. Future approaches are likely to simultaneously target ovarian cancer and supporting cells, such as M2 macrophages, MDSCs, CAFs, MSCs, CSCs, and ECs with drug-loaded NPs.

**Figure 4:**
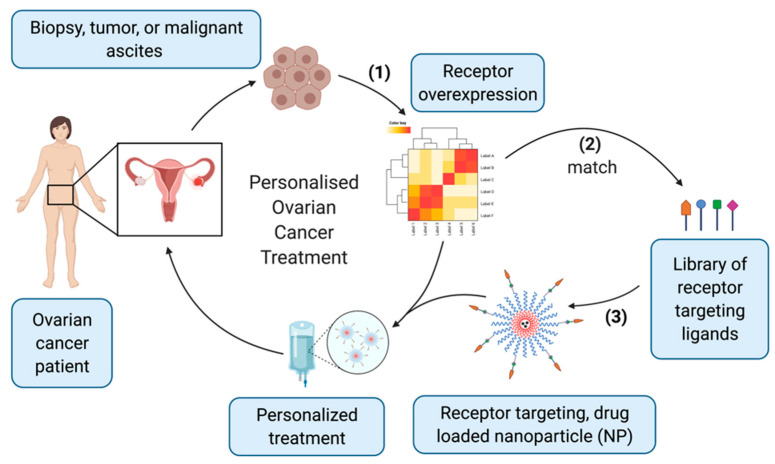
Schematic of personalized ovarian cancer treatment, showing (**1**) analysis of tumor biopsies for concurrently overexpressed GPCRs, (**2**) matching of receptor targeting ligands guided by receptor expression profiles, and (**3**) design of NPs with self-assembling polymers furnished with targeting ligands. Created with BioRender.com.

Gene expression signatures can be used to systematically identify targeting receptors to specifically destroy supporting immune cells, stromal cells, or neo-vasculature components along with malignant tumor cells and CSCs. An unprecedented analysis of gene expression and proteomics meta-data can be used to systematically discover overexpressed GPCRs as novel cell surface biomarkers on ovarian cancer cells. Provided the genetic heterogeneity of ovarian cancers, it is unlikely that a single receptor or biomarker would be appropriate for use as a molecular target for all patients. A detailed understanding of the differential targeting receptor expression landscape between cancer patients will lay the foundations for personalized ovarian cancer cell targeting. To achieve optimal drug targeting and therapeutic benefit, personalized, adaptable platforms must be developed that can express a variety of different ligands based on patient-specific receptor overexpression. Ideally, this would be achieved through (1) identification of receptor overexpression from patient biopsies; (2) determination of appropriate targeting vectors using a library of ligands for the identified receptors; and (3) incorporation of the targeting ligand onto the drug-loaded NP surface (Figure 4). In practice, this could be accomplished through the development of modular and highly adaptable NP platforms, that could be interchangeably furnished with different targeting ligands using a biorthogonal ligation approach, allowing the NPs to be tailored to the patient. Innovation of novel drug delivery platforms is ongoing through the culmination of several elements of experimental design, validation, and engineered chemical devices to establish this novel personalized treatment paradigm.

An interesting option could also be the targeting of two receptors per cell, delivering lethal combinations of anti-cancer agents (e.g., chemotherapeutic agent combinations or in combination with targeted therapies, such as PARP or kinase inhibitors). This will introduce a novel way to increase the therapeutic index if the targeting receptors are selected to avoid dual expression in healthy tissues.

While a variety of receptors can be exploited for active-targeting purposes, GPCRs present as an attractive target for the design of effective ovarian cancer-specific NP drug delivery platforms, with many being frequently overexpressed in ovarian cancers compared to healthy tissues. While GPCR targeting holds significant promise in the development of ovarian cancer delivery systems, this strategy is yet to be further explored as only a handful of GPCRs have presently been investigated for this purpose, highlighting this as a potential avenue toward case-by-case personalized ovarian cancer treatment. These novel concepts have massive potential to develop safe and personalized ovarian cancer cures. Preclinical proof of principle for these approaches can be provided in relevant animal xenograft models.

## Figures and Tables

**Table 1:** List of FDA approved nanomedicines for cancer treatment.

Carrier System	Nanomedicine (Drug/Mechanism)	Size (nm)	Targeted Cancer	Result
Liposome	Doxil/ Caelyx™ (Doxorubicin)	80–100	Karposi’s Sarcoma, multiple myeloma, Ovarian and metastatic breast cancer	Reduces the toxicity of DOX and remains longer in the blood stream [81,85]
	Myocet (Doxorubicin)	100–250	Breast cancer	Reduces the cardiotoxicity of DOX while maintaining its anti-tumor efficacy [82,86]
	DaunoXome (Daunorubicin)	45–80	Karposi’s sarcoma	Protects DOX from enzymatic and chemical degradation, avoids its uptake by normal tissues [86]
	DepoCyt (Cytarabine)	20	Lymphomatous meningitis	Helps in slow and targeted release of cytarabine [86]
	Marqibo (Vincristine)	100–115	Acute Lymphoblastic Leukemia	Overcomes the pharmacokinetic and dosage limitation of vincristine [87]
	Onivyde (Irinotecan)	80–140	Pancreatic cancer	Liposomes are accumulated in the tumor leading to slow release of drug, allowing the drug to act longer [88]
	Vyxeose (Daunorubicin and cytarabine)	100	Acute myeloid leukemia	Liposomes are engulfed by tumor cells to a greater extent than the normal cells hence, increasing the survival rate [88]
	Lipusu (Paclitaxel)	400	NSCLC, ovarian, and breast cancer	Changes the biodistributions and reduces the toxicity in the system [89,90]
	Lipodox (Doxorubicin hydrochloride)	20	Breast and ovarian cancer	Increased stability in blood stream and can enter the altered and compromised vasculature of tumors [91,92]
Albumin	Abraxane (Paclitaxel)	130	NSCLC, Breast/Pancreatic cancers	Delivers high concentrations of the drug to the cancer cells and reduces the rate of side effects [87,93,94]
Polymeric	Oncaspar (L-asparaginase)	130	Acute lymphoblastic leukemia	Has longer half-life, lowers the drug level in blood cancer cells and stops the cancer from growing. It also has slower clearance than asparaginase [95]
	Eligard (Leuprolide acetate)	30–100	Prostate cancer	Able to deliver leuprolide acetate at a controlled rate over a one-, three-, four- or six-month therapeutic period [88]
Micelles	Nanoxel (Paclitaxel)	80–100	Metastatic breast cancer	Decreases toxicity, increases the antitumor activity due to the selective accumulation of the drug in tumor cells [96]
	Genexol PM (Paclitaxel)	20–50	NSCLC, breast and ovarian cancer	Allows increased dose of paclitaxel with improved efficacy and without compromising the safety of patients [97]
Iron Oxide	Feridex (Ferumoxides)	162–173	MRI contrast agent for detection of liver metastasis	These are easily taken up by cells of RES system, hence helps in detecting tumor cells [98,99]
	Nanotherm (Hyperthermal)	15	Glioblastoma, prostate cancer	Reduce the risk of overtreatment and effectively differentiates between tumors and healthy cells [87]
